# Phenotypic Variation in Flower Color and Morphology in the Gerbera (*Gerbera hybrida*) F_1_ Hybrid Population and Their Association with EST-SSR Markers

**DOI:** 10.3390/ijms25010203

**Published:** 2023-12-22

**Authors:** Yiwei Zhou, Xinru Zou, Fulong Yan, Jingjuan He, Sixian Zeng, Yunyi Yu, Xiaoshuang Tang, Xuanguo Liang, Xiuping Cai, Rangcai Yu, Yanping Fan

**Affiliations:** 1 The Research Center for Ornamental Plants, College of Forestry and Landscape Architecture, South China Agricultural University, Guangzhou 510642, China; zhouyiwei@gadds.cn (Y.Z.); hejingjuan@stu.scau.edu.cn (J.H.); rcyu@scau.edu.cn (R.Y.); 2 Guangdong Key Lab of Ornamental Plant Germplasm Innovation and Utilization, Environmental Horticulture Research Institute, Guangdong Academy of Agricultural Sciences, Guangzhou 510642, China; 3 College of Life Sciences, South China Agricultural University, Guangzhou 510642, China; 4 Guangdong Key Laboratory for Innovative Development and Utilization of Forest Plant Germplasm, South China Agricultural University, Guangzhou 510642, China

**Keywords:** gerbera, genetic analysis, flower color, association analysis

## Abstract

Gerbera (*Gerbera hybrida*) is a widely cultivated ornamental plant. However, its genetic improvement is limited by the lack of genetic analysis and molecular markers for traits. In this study, we analyzed the phenotypic and genotypic variation of 140 F_1_ progeny from two gerbera varieties with different flower types and colors. We evaluated the flower’s morphology, color, and pigment content of the F_1_ population and performed cluster principal component analysis (PCA) and correlation analysis. The results showed that the main ornamental traits of the hybrid progeny varied greatly. The segregation ratios of single and double flowers and ligulate and split ray florets were both 1:1. The flower colors of the F_1_ progeny were mainly red and purple-red, similar to the male parent’s color. Furthermore, we conducted a genetic analysis of the hybrid progeny using EST-SSR markers and performed association analysis with phenotypic traits. We identified 2, 2, 3, 1, and 2 loci to be associated with peduncle length (PL), ray floret length (RFL), and outer ray floret; the level of apex relative to the top of involucre (LAI); outer corolla lips (OCL); and the b* of ray floret color, respectively. Our results reveal the genetic patterns of important ornamental traits and provide a theoretical basis and practical tools for gerbera genetic breeding.

## 1. Introduction

Gerbera (*Gerbera hybrida*), a perennial herb of the Asteraceae family, is extensively utilized in the production of cut flowers, potted plants, and landscape gardening due to its substantial ornamental and economic value [1,2]. In 2020 alone, the sales value of cut gerbera sold by Royal FloraHolland reached EUR 130 million (https://www.statista.com/statistics/829413/sales-value-of-cut-flowers-sold-by-royal-floraholland-by-type/, accessed on 2 August 2023). The color and morphology of the flowers are key ornamental traits of gerbera. The flowers display a broad spectrum of colors, including white, yellow, orange, pink, red, and purple, and exhibit various unique morphologies, such as the patterning of the ray, disk florets, and a distinctive petal trait known as “split ray floret”. This diversity positions gerbera as an ideal model for studying flower color and morphology [3,4,5,6,7,8,9,10]. However, research on the color and split ray floret traits of gerbera is currently limited, which restricts the directional breeding of this species.

Gerbera is derived from the crossbreeding of two wild African species, *G. jamesonii* and *G. viridifolia* [11]. Previous studies investigated the genetic basis of some important ornamental traits of gerbera. Kloos et al. [11] found that the split ray floret gerbera phenotype is controlled by a single dominant gene (*Sp*). When two ligulate ray floret gerberas are crossed, no split ray floret offspring are produced. When both parents are split ray floret gerberas, the offspring ratio of the split ray to ligulate ray florets is 3:1. When one parent is a spider gerbera, the offspring ratio of the split ray to ligulate ray florets is either 1:0 or 1:1, and the F_2_ generation ratio is 3:1 [11]. The mechanism of the split petal formation is still unknown. One possibility is that it may result from incomplete petal fusion. Kotilainen et al. [12] reported that the tongue-shaped flowers of normal small-flowered gerbera are formed by the fusion of three petal fragments. Kumar evaluated 13 gerbera genotypes for 15 quantitative traits and observed significant genotypic variation, a high genotype–environment interaction, and high heritability and genetic gain for most traits [13]. The traits with high heritability and genetic gain included the leaf number, leaf size, flowering time, disc diameter, flower-stalk length, ray floret number, and ray floret size. Rymbai’s study [14] assessed 37 gerbera accessions for yield and quality traits and detected high genetic diversity, heritability, and genetic gain among them. However, these studies mainly involved different varieties and small population sizes. Moreover, there is a lack of systematic genetic analysis of flower color, the split ray floret, and other important ornamental traits, which limits the comprehensive genetic improvement of gerbera.

Molecular marker-assisted selection (MAS) can accelerate directional breeding by developing molecular markers that are linked to specific traits [15,16]. Previous studies have used RAPD [17], DAMD [18], ISSR [18], and SSR [19,20,21] markers to evaluate the genetic diversity of gerbera. However, few studies have focused on molecular markers related to important ornamental traits of gerbera. Since gerbera has no published genome sequence, SSR molecular markers, which are highly polymorphic [22], remain essential for gerbera breeding research. In our previous work, we developed new EST-SSR markers based on transcriptomes and identified several molecular markers linked to flower color through association analysis, laying the foundation for the molecular marker breeding of gerbera [21]. Fu et al. [23] and Bhattarai et al. [24] also developed SNP markers and constructed a genetic map of gerbera. However, the high heterozygosity of gerbera limited the mapping of SNPs to linkage groups. Developing molecular markers related to a wider range of important traits is imperative to meet the breeding requirements of gerbera varieties, which have diverse traits.

**Figure 1:**
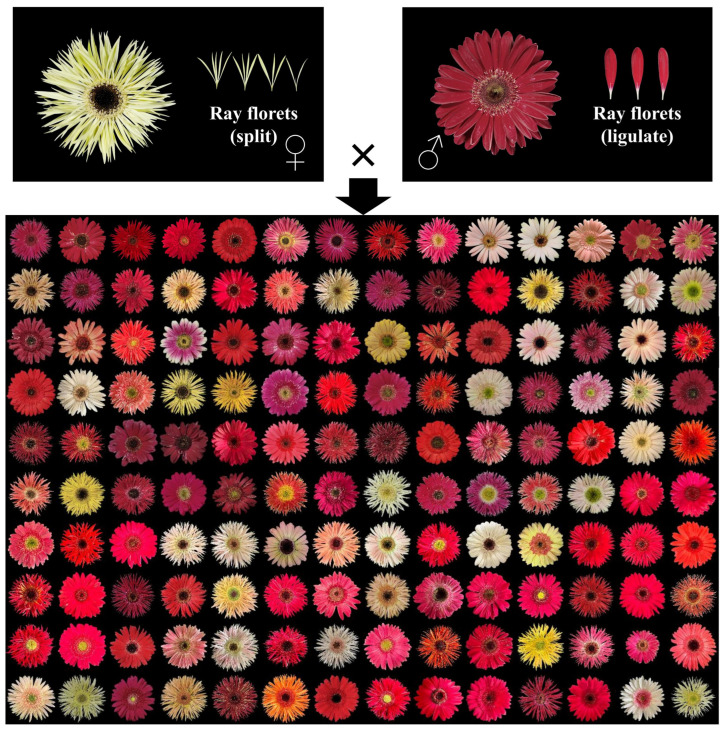
Flower heads of ‘LSB’ (♀), ‘GMZ’ (♂) and their 140 F_1_ hybrid progeny.

Currently, the breeding of new gerbera varieties still relies on conventional methods. Therefore, understanding the genetic basis of gerbera traits and developing molecular markers associated with them are of great significance for accelerating the breeding process of gerbera. In this study, we measured the phenotypic and analyzed the genetics of 12 key ornamental traits in 140 F_1_ hybrid progeny, which were obtained via crossing the split ray floret gerbera variety ‘LSB’ (female parent) and the ligulate ray floret gerbera variety ‘GMZ’ (male parent). The traits included peduncle length (PL), peduncle diameter (PD), flower head height (FHH), flower head diameter (FHD), ray floret length (RFL), disc diameter (DiD), ray floret number (RFN), and outer ray floret; the level of the apex relative to the top of involucre (LAI); flower type (FT); dark disc (DaD); outer corolla lips (OCL); and ray floret color (RFC). Figure 1 shows the flower head of the parents and all the progenies. We also evaluated the CIELab* parameters, the total anthocyanin content (AT), and total carotenoid content (CT) and examined their correlation with flower color. In addition, we developed SSR molecular markers for these traits. This research provides insights into the molecular mechanisms underlying the formation of major ornamental traits and offers a theoretical basis for early selection in gerbera hybrid breeding.

## 2. Results

### 2.1. Genetic Analysis of Morphological Traits and Grouping Colors

The variation in the seven numerical morphological traits was assessed by the coefficient of variation (CV), which ranged from 13.05% to 23.11% across all hybrid individuals. Peduncle length showed the highest variation, while the flower head diameter showed the lowest variation (Tables S1 and S2). The F_1_ individuals tended to have a larger peduncle diameter and flower head diameter than the parent plants, with 62.14% and 49.29% of the total accessions, respectively. On the other hand, the F_1_ individuals tended to have a smaller disc diameter and ray floret number than the parent plants, with 57.86% and 51.43% of the total accessions, respectively (Table S2). Additionally, most of the hybrid offspring had a peduncle length (97.14%), flower head height (82.86%), and ray floret length (63.57%) within the parental range.

**Figure 2:**
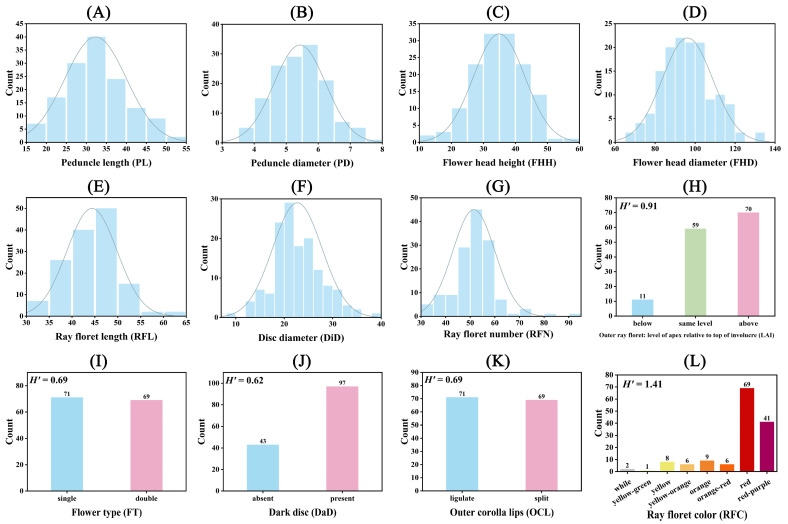
Diversity analysis of 12 phenotypic traits of the F_1_ hybrid population. “*H*” is the Shannon–Wiener index of genetic diversity. The count of this category is indicated by the numbers above the bars. (**A**) Peduncle length (PL); (**B**) peduncle diameter (PD); (**C**) flower head height (FHH); (**D**) flower head diameter (FHD); (**E**) ray floret length (RFL); (**F**) disc diameter (DiD); (**G**) ray floret number (RFN); (**H**) outer ray floret; the level of the apex relative to top of the involucre (LAI); (**I**) flower type (FT); (**J**) dark disc (DaD); (**K**) outer corolla lips (OCL); and (**L**) ray floret color (RFC).

The numerical traits’ distribution among the hybrid offspring and parent plants is depicted in Figure 2. The peduncle length varied from 15.91 cm to 51.65 cm, with only four hybrid offspring outside the parental range (Figure 2A and Table S2). The peduncle diameter ranged from 3.82 mm to 7.85 mm, with the majority of the hybrid offspring (87) surpassing the parent plants’ values (Figure 2B and Table S2). The flower head height spanned from 14.24 mm to 57.34 cm, with most of the hybrid offspring (116) falling within the parental range (Figure 2C and Table S2). Regarding the flower head diameter, 62 hybrid offspring were within the parental range, while 69 exceeded it (Figure 2D and Table S2). For the ray floret length, 89 hybrid offspring were within the parental range, with 50 exceeding it (Figure 2E and Table S2). For the disc diameter, 81 hybrid offspring had values lower than the parents; 29 were within the parental range, and 30 exceeded it (Figure 2F and Table S2). In terms of the ray floret number, 72 hybrid offspring had values lower than the parents, 61 exceeded the parental values, and only 7 were within the parental range (Figure 2G and Table S2).

Significant disparities were observed between the two parent plants in terms of LAI, flower type, outer corolla lips, and ray floret color, with the dark disc being the sole consistent trait (Table S3). The Shannon–Wiener index (*H*), calculated for the five descriptive traits of F_1_ hybrids, varied from 0.62 to 1.41 (Table S3). For LAI, ‘LSB’ was classified as above and ‘GMZ’ as below, while the hybrid offspring exhibited the following three categories: below (11), the same level (59), and above (70), representing 7.86%, 42.14%, and 50.00% of the total hybrid offspring, respectively (Figure 2H and Table S3). For flower type, ‘LSB’ was double, ‘GMZ’ was single, and among the hybrid offspring, 71 were double, and 69 were single (Figure 2I and Table S3). For the dark disc trait, both parents were categorized as present, while among the hybrid offspring, 43 were absent, and 69 were present (Figure 2J and Table S3). For outer corolla lips, ‘LSB’ was split, ‘GMZ’ was ligulate, and among the hybrid offspring, 71 were ligulate, and 69 were split (Figure 2K and Table S3). For the ray floret color, ‘LSB’ was white, ‘GMZ’ was red-purple, and the hybrid offspring could be categorized into eight colors: white (1), yellow-green (1), yellow (8), yellow-orange (6), orange (9), orange-red (6), red (69), and red-purple (41), with red and red-purple accounting for the majority (78.57%) (Figure 2L and Table S3).

Building on Kloos’s research [11], we conducted a chi-square test analysis for flower type, dark disc, and outer corolla lips (Table S4). For the flower type trait, we hypothesized that the genotype of ‘LSB’ was *Crcr* and that of ‘GMZ’ was *crcr*. The chi-square test analysis showed that the segregation ratio of the hybrid offspring was consistent with the expected 1:1 ratio (Table S4). For the dark disc trait, we hypothesized that the genotypes of both ‘LSB’ and ‘GMZ’ were *Dddd*. When the genotype was *Dddd* or *DdDd*, a dark disc was present, and when the genotype was *dddd*, a dark disc was absent. The chi-square test analysis showed that the segregation ratio (present/absent) of the hybrid offspring was consistent with the expected 3:1 ratio (Table S4). For the outer corolla lips trait, we hypothesized that the genotype of ‘LSB’ was *Spsp* and that of ‘GMZ’ was *spsp*. The chi-square test analysis showed that the segregation ratio of the hybrid offspring was consistent with the expected 1:1 ratio (Table S4).

The correlation analysis revealed 19 pairs of traits that were significantly correlated among the seven numerical and five descriptive traits (Figure S1). Peduncle length (PL) was significantly positively correlated with the ray floret length (RFL) and leaf area index (LAI). Peduncle diameter (PD) was significantly positively correlated with the flower head diameter (FHD), RFL, disc diameter (DiD), and ray floret number (RFN). The flower head height (FHH) was significantly positively correlated with FHD, RFL, LAI, dark disc (DaD), and outer corolla lips (OLC). FHD exhibited a significant positive correlation with the RFL and RFN. RFL was significantly positively correlated with RFN and LAI. LAI and OLC showed a significant positive correlation. Additionally, flower type (FT) was significantly negatively correlated with FHH and OLC, and OLC was significantly negatively correlated with ray floret color (RFC).

### 2.2. Clustering and Principal Component Analysis Based on 12 Phenotypic Traits

**Figure 3:**
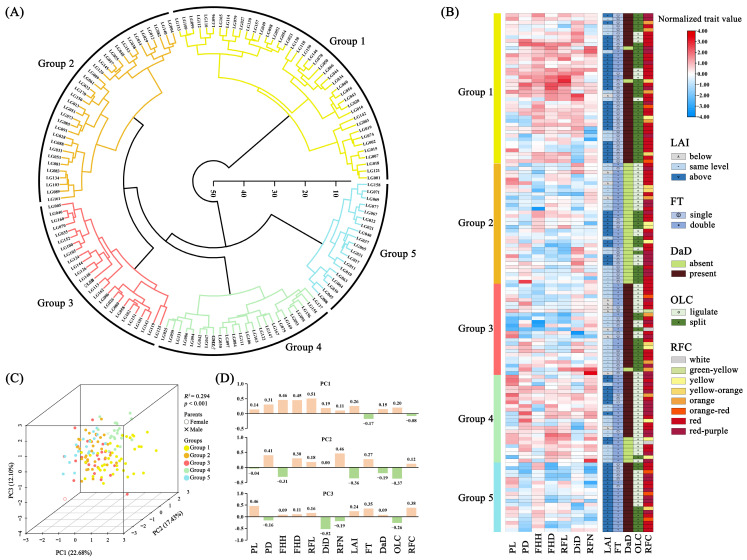
Hierarchical cluster analysis (HCA) and principal component analysis (PCA) of gerbera’s 12 phenotypic traits. (**A**) HCA tree of two parents and 140 F_1_ hybrid progeny; (**B**) Comparison of 12 trait performances among five groups based on HCA analysis; (**C**) 3D PCA score plot, and (**D**) loading plots of 12 phenotypic traits on the first three PCAs.

Hierarchical cluster analysis (HCA) revealed that the two parent plants and 140 F_1_ individuals could be classified into five groups based on 12 phenotypic traits (Figure 3A). Group 1 comprised 41 individuals with the largest values of FHH, FHD, RFL, DiD, and 82.93% of spider gerberas (Figure 3B). Group 2 consisted of 33 individuals lacking a dark disc (Figure 3B). Group 3 included ‘LSB’ and 24 F_1_ individuals with the smallest values of PL and FHH, the highest proportion below the status in LAI, and lighter ray florets (Figure 3B). Group 4 contained ‘GMZ’ and 23 F_1_ individuals with the largest values of PL, PD, RFN, and the ratio of double flower (Figure 3B). Group 5 consisted of 19 F_1_ individuals with the smallest values of PD, FHD, and RFN, above the status in LAI and darker ray florets (Figure 3B).

The first, second, and third principal components (PCs) accounted for 22.68%, 17.43%, and 12.10% of the overall variability, respectively (Figure 3C). The permutational multivariate analysis of variance (PERMANOVA) confirmed that the results of the five groups were significant (*p* < 0.001), and the *R^2^* was 0.294 (Figure 3C and Table S5). According to the PC1 loading plot, 10 traits had positive coefficients, while only FT and RFC had negative values (Figure 3D). In the PC2 loading plot, seven traits had positive coefficients, and five had negative coefficients (Figure 3D). The PC3 loading plot had eight traits with positive coefficients and four with negative coefficients (Figure 3D).

### 2.3. Colorimeter Determination and Pigment Diversity Analysis

**Figure 4:**
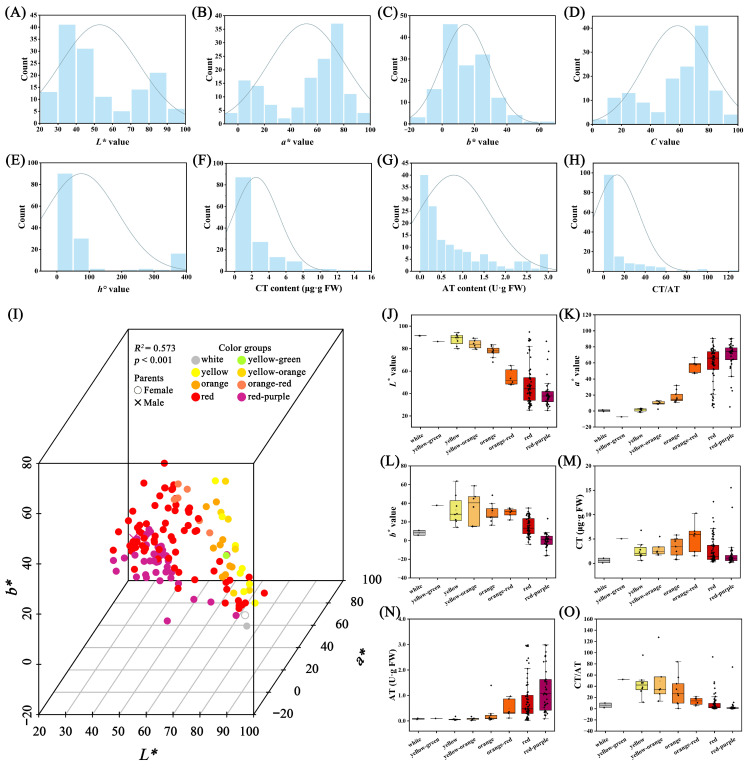
Diversity analysis of parameters detected by colorimeter and flower pigments in the F_1_ hybrid population. Frequency distribution histogram of the L* value (**A**), a* value (**B**), b* value (**C**), *C* value (**D**), *h°* value (**E**), CT content (**F**), AT content (**G**), and CT/AT (**H**). Three-dimensional Scatter Chart of L*, a*, and b* (**I**) and comparison of L* (**J**), a* (**K**), b* (**L**), CT (**M**), AT (**N**), and CT/AT (**O**) for the eight color groups.

The distribution of the five parameters detected by the colorimeter, CT content, AT content, and CT/AT among the hybrid offspring and parent plants is presented in Figure 4A–H and Table S6. The L* value ranged from 24.96 to 94.79, with most hybrid offspring (129) falling within the parental range (Figure 4A and Table S6). The a* value spanned from −7.29 to 90.71, with most hybrid offspring (105) within the parental range (Figure 4B; Table S6). The b* value varied between −16.23 and 63.66, with 58 hybrid offspring having values lower than the parents and 72 exceeding the parental values (Figure 4C and Table S6). For the *C* value, 105 hybrid offspring were within the parental range, while 33 exceeded it (Figure 4D and Table S6). In the case of the *h°* value, 86 hybrid offspring were within the parental range, with 23 exceeding it (Figure 4E and Table S6). The CT content ranged from 0.11 μg·g FW to 94.79 μg·g FW, with 53 hybrid offspring having values lower than the parents, 43 within the parental range, and 44 exceeding it (Figure 4F and Table S6). The AT content varied from 0.02 U·g FW to 2.99 U·g FW, with 31 hybrid offspring having values lower than the parents, 101 within the parental range, and 8 exceeding it (Figure 4F and Table S6). Regarding the CT/AT ratio, 35 hybrid offspring had values lower than the parents, 63 exceeded the parental values, and only 42 were within the parental range (Figure 4G and Table S6).

The ray floret color of the F_1_ population can be divided into eight groups according to the RHS color chart: white, yellow-green, yellow, yellow-orange, orange, and orange-red, red, and redddish-purple. The L*, a*, b* values measured via the colorimeter were displayed in a 3D scatter plot, showing clear separation among the individuals of different color groups. The PERMANOVA result confirmed that the grouping result of the eight color groups was significant (*p* < 0.001) with an *R^2^* of 0.573 (Figure 4I and Table S7). The L* value indicated the lightness of the color, and it was higher in the white, yellow-green, yellow, yellow-orange, orange, and orange-red color groups and lower in the orange-red, red, and red-purple color groups (Figure 4J). The a* value was lower in the white, yellow-green, yellow, yellow-orange, orange, orange-red color groups and higher in the orange-red, red, and red-purple color groups (Figure 4K). The b* value was lower in the white-red-purple groups and higher in the other six color groups (Figure 4L). We also measured the total carotenoid (CT) and total anthocyanin (AT) content of the ray floret. The CT content was lower in the white and red-purple groups and higher in the other six color groups (Figure 4M). The AT content was lower in the white, yellow-green, yellow, yellow-orange, and orange groups and higher in the orange-red, red, and red-purple color groups (Figure 4N). The CT/AT ratio indicated the balance of these two pigments, and it was lower in the white, orange-red, red, and red-purple color groups and higher in the yellow-green, yellow, yellow-orange, and orange color groups (Figure 4O). The correlation analysis showed that the CT content was significantly positively correlated with the b* value, the AT value was significantly positively correlated with the a* and *C* values, and significantly negatively correlated with the L* and b* values (Figure S2). The CT/AT value was significantly positively correlated with the L*, b*, and CT content and significantly negatively correlated with the a*, *C*, and AT content.

### 2.4. Clustering and Principal Coordinate Analysis Based on EST-SSR Markers

**Figure 5:**
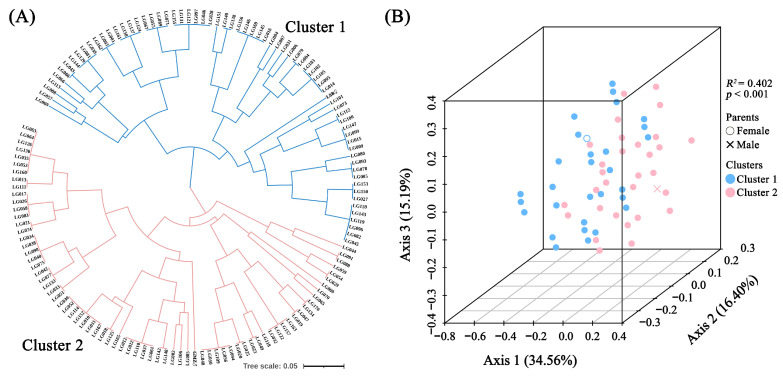
UPGMA cluster (**A**) and PCoA (**B**) analysis of parents and 140 hybrid progenies based on SSR genotype data.

We performed the Unweighted Pair Group Method with Arithmetic Mean (UPGMA) cluster analysis and Principal Coordinate Analysis (PCoA) based on EST-SSR marker data (Figures S3–S7). The UPGMA analysis divided the two parent plants and 140 hybrid offspring into two clusters (Figure 5A). Cluster 1 included ‘LSB’ and 68 F_1_ hybrid offspring, while Cluster 2 comprised ‘GMZ’ and 72 hybrid offspring. The PCoA analysis also supported the clustering results of the UPGMA analysis, and the first, second, and third axes accounted for 34.56%, 16.40%, and 15.19% of the overall variability, respectively (Figure 5B). PERMANOVA analysis confirmed that the grouping result of the two clusters was significant (*p* < 0.001) with an *R^2^* of 0.402 (Table S8).

### 2.5. Association Analysis between Traits and Molecular Markers

**Figure 6:**
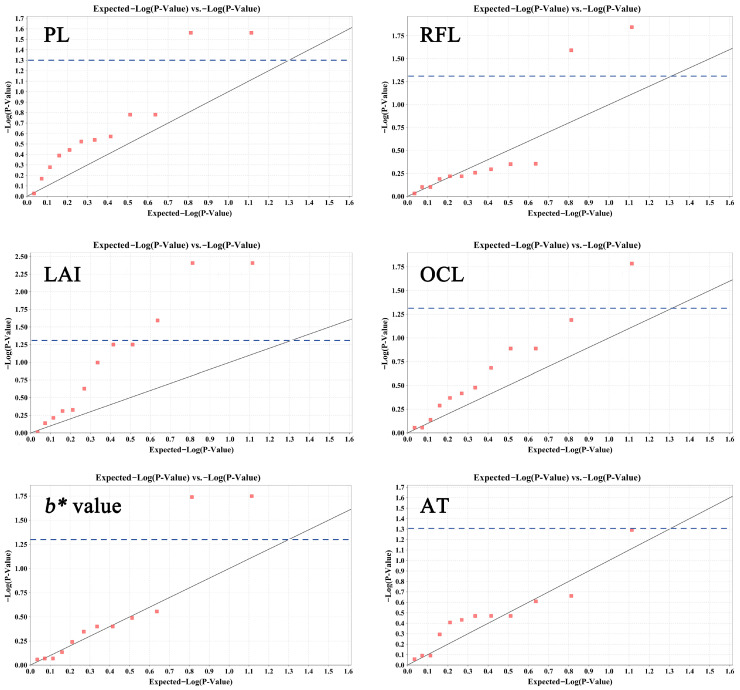
Q–Q plot of the SSR-based association analysis for six traits with significantly associated loci. Values above the blue dashed line indicate *p* < 0.05.

We examined the molecular markers linked with primary ornamental traits using EST-SSR genotyping. A general linear model (GLM, K model) analysis indicated that 10 SSR loci were significantly associated with five traits (*p* < 0.05) (Table S9 and Figure 6). Two, two, three, one, and two loci were significantly associated with PL, RFL, LAI, OCL, and b*, respectively. Additionally, one locus associated with AT had a *p*-value of 0.0512 and was also included in the analysis. These 11 loci explained 2.73~5.96% of the phenotypic variation (Table S9 and Figure 6).

**Figure 7:**
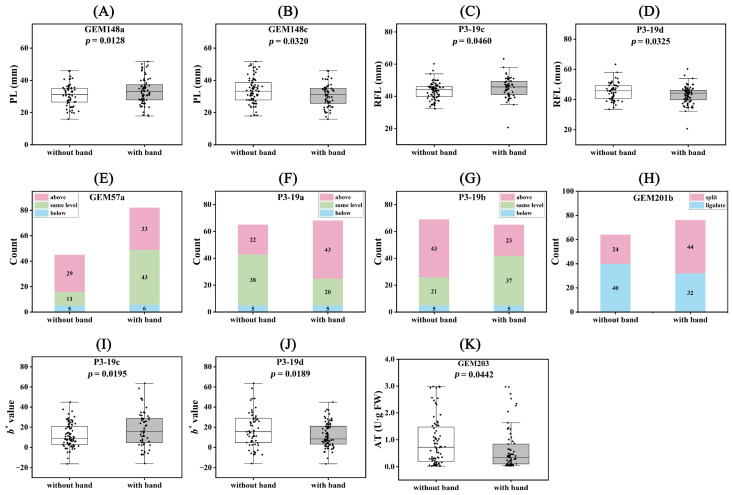
Comparison of population phenotypes for significant association loci. (**A**) GEM148a and PL; (**B**) GEM148c and PL; (**C**) P3-19c and RFL; (**D**) P3-19d and RFL; (**E**) GEM57a and LAI; (**F**) P3-19b and LAI; (**G**) P3-19a and LAI; (**H**) GEM201b and OCL; (**I**) P3-19c and b*; (**J**) P3-19d and b*; (**K**) GEM203 and AT.

We performed Student’s *t*-test on the phenotypes of these differential loci, and all numerical traits showed significant differences (Figure 7). For the LAI trait, the GEM57a locus showed that 64.44% of individuals without bands were in the ‘above’ state, while 51.81% of individuals with bands were in the ‘same level’ state. Similarly, at the P3-19b locus, 62.32% of individuals without bands and 56.92% of individuals with bands were in the ‘above’ and ‘same level’ states, respectively. At the P3-19a locus, 58.46% of individuals without bands were in the ‘same level’ state, and 63.24% of individuals with bands were in the ‘above’ state. For the OFL trait at the GEM201b locus, 62.50% of individuals without bands were in the ‘ligulate’ state, and 56.41% of individuals with bands were in the ‘split’ state.

## 3. Discussion

In this study, we first analyzed the variation and inheritance patterns of 12 phenotypic traits in the F_1_ population of ‘LSB’ and ‘GMZ’. Among them, PD, FHH, FHD, and RFL had positive mid-parent heterosis values, while PL, DiD, and RFN had negative mid-parent heterosis values. In addition, the F_1_ population showed trait segregation for LAI, FT, DiD, OCL, and RFC, especially RFC, with a high Shannon–Weaver index of 1.41. The segregation ratio of ligulate and split in OCL was 1:1, corroborating with the analysis result of the inheritance patterns of other gerbera varieties [11]. Additionally, we analyzed the inheritance pattern of dark flower hearts. The chi-square test showed that their segregation ratio followed 3:1, suggesting that it is likely controlled by a single dominant gene, as light-colored flower hearts appeared in the offspring when both parents had dark flower heart phenotypes. This needs to be verified in additional hybrid populations.

Investigating the principles of genetics is an essential phase in the process of floral genetic breeding. Many ornamental plants are hybrids, leading to extensive trait segregation typically observed in F_1_ hybrids. Understanding the inheritance patterns of key ornamental traits can provide insights into more effective genetic breeding. The inheritance patterns of ornamental traits in various plants, such as *Streptocarpus* [25], *Lilium* [26], *Chrysanthemum* [27,28,29,30], and *Hedychium* [31,32], have been examined. The study of floral trait inheritance patterns in Asteraceae ornamental plants is a popular research topic, with the inheritance patterns of several traits, such as flower type, being clarified [29,30]. We conducted a genetic analysis of the primary ornamental traits of the F_1_ hybrid progeny of ‘LSB’ and ‘GMZ’. Initially, all quantitative traits exhibited a continuous distribution. For flower color, paternal inheritance was more dominant, with 85% of individuals showing red or purple-red hues. Interestingly, OCL had a significant negative correlation with FT and RFC, indicating that when the offspring had a split OCL, they were more likely to have single-petaled FT and light-colored RFC. In addition to analyzing the inheritance patterns of different traits, we also applied multivariate statistical analysis methods, such as HCA and PCA, to analyze the phenotypic traits. We successfully divided 140 hybrid offspring and their parents into five groups based on 12 phenotypic traits, and the PERMANOVA analysis revealed that the grouping result was significant, which further suggests that hybridization greatly diversifies the overall phenotypic trait types of gerberas.

Flowering plants exhibit a remarkable diversity of flower types and colors, which result from the synthesis of various pigments [33,34]. Carotenoids are mainly responsible for yellow and orange hues, while anthocyanins produce colors ranging from red to purple [33,35]. We found that as the color deepened, the AT value gradually increased, and the CT/AT value gradually decreased. RFC had a significant positive correlation with AT and a significant negative correlation with CT/AT. The CT value was higher in the yellow-green, yellow, yellow, orange, and orange groups, corroborating with previous research results. In previous research, we analyzed the petals of 123 distinct gerbera varieties. PLS-DA analysis revealed that AT primarily influenced the formation of the red and purple color groups, and CT predominantly affected the formation of the orange and yellow color groups [21]. In potted multiflora chrysanthemum, 273 varieties were divided into six color groups, with anthocyanins identified only in the pink, purple, orange-brown, and red color groups. All color groups contained carotenoids, but the red and orange-brown groups had a significantly higher content [36]. We also found that the L* value had a significant negative correlation with AT, the a* value had a significant positive correlation with AT, and the b* value had a significant positive correlation with CT. L* and a* values were significantly correlated with anthocyanins, and the b* value is significantly correlated with carotenoids in many color phenotype studies, especially in our previous study on different gerbera varieties, where we obtained similar results [21]. These results suggest that L* and a* values can be used as indicators to estimate AT values, and b* values can be used as indicators to estimate CT values, regardless of different gerbera varieties or F_1_ hybrid populations. This provides an effective method for the rapid evaluation of CT and AT values in gerbera ray florets.

Association analysis is a pivotal approach for identifying molecular markers linked to specific traits, and SSR markers are integral in horticultural plants [37,38]. These markers have been effectively used to identify significant association markers in various ornamental plants, such as caladium [39], chrysanthemum [40], *Hemerocallis* [41], *Paeonia rockii* [42], and *Syringa oblata* [43]. We employed five previously developed SSR markers to analyze 19 indicators of the gerbera F_1_ population and found significant associations between 2, 2, 3, 1, and 2 loci with PL, RFL, LAI, OCL, and b*, respectively. These markers offer new insights for the molecular marker-assisted breeding of gerbera, especially in expanding populations of ‘LSB’ and ‘GMZ’. These markers explained 3.48% to 5.96% of phenotypic variances, which could be attributed to the influence of polygenes on most traits. The *p*-value of the association between AT value and GEM203 was 0.0512, and the phenotypic variance-explained (*R^2^*) value was only 2.73%. A *t*-test indicated that the mean AT value differed significantly between the without-band and with-band individuals. Previous studies have shown that *R^2^* is often low in association with the analysis of complex quantitative traits, especially in heterozygous plants such [44,45], and gerbera is a typical heterozygous plant. SNP markers have been developed in recent years, but only a small fraction can be mapped to linkage groups, possibly because of the high heterozygosity of gerbera [23,24]. The lack of a genome further limits the molecular marker-assisted breeding of gerbera. Therefore, it accelerates the sequencing and development of molecular markers for the gerbera genome to establish a foundation for its genetic breeding.

## 4. Materials and Methods

### 4.1. Plant Materials

The female parent ‘LSB’, the male parent ‘GMZ’, and their 140 F_1_ hybrid offspring were grown under the same conditions at South China Agricultural University, Guangzhou, China (23.16° N, 113.36° E). The controlled greenhouse provided a constant environment for the plants, with a temperature of 26 ± 2 °C and a relative humidity of 75–80%. The cross was completed through emasculation, bagging, and multiple pollination in eight flower heads of the male. We obtained about 170 seeds in total, which were sown in a constant temperature incubator (26 ± 1 °C). After seedling and potting, we finally obtained 140 flowering offspring and performed subsequent analyses. All samples were collected and analyzed at the flowering stage. The samples were immediately frozen in liquid nitrogen and stored at −80°C for pigment analysis and DNA extraction. Detailed information on the accessions is provided in Table S1.

### 4.2. Observation of Morphological Traits

We measured 11 morphological traits of the parents and F_1_ hybrids following the DUS test guidelines for gerbera (UPOV TG/77/9). These traits were the peduncle length (PL), peduncle diameter (PD), flower head height (FHH), flower head diameter (FHD), ray floret length (RFL), disc diameter (DiD), ray floret number (RFN), outer ray floret; the level of the apex relative to the top of involucre (LAI); flower type (FT); dark disc (DaD); and outer corolla lips (OCL). We used a ruler to measure PL, PD, FHH, FHD, RFL, DiD, and RFN. We visually assessed LAI, FT, DaD, and OCL according to the categories based on the guidelines. We observed all traits at the flowering stage. We took 10 measurements for each trait as replicates for each accession.

### 4.3. Determination of Flower Color Traits

We recorded the color group and the number of each accession (two parents and 140 F_1_ hybrids) using the RHS color chart and assigned a code to each color group. We determined the ray flower color (RFC) according to the RHS color group. We measured the main color of the ray floret with a CS-210 precision colorimeter (Hangzhou Caipu Technology, Hangzhou, China). We measured the CIELab* color coordinates (lightness, L*; chromatic components, a* and b*) and calculated the hue angle (*h°*) and the chroma (*C*) using the formulas *h°* = arctan (b*/a*), C = (a*^2^ + b*^2^)^1/2^ [46]. We took 10 measurements for each parameter as replicates for each accession.

### 4.4. Analysis of Total Carotenoid Content and Anthocyanin Contents

We followed Zhou et al. [21] with minor modifications to measure the total carotenoid (CT) and total anthocyanin (AT) contents. For CT, we extracted 2 g of fresh flowers in 10 mL of acetone and centrifuged the mixture at 5000 rpm for 8 min. We repeated this process until the supernatant was clear. We added 15 g of anhydrous sodium sulfate to the extract in a 50 mL flask. The absorbance (A) was measured at A450. We used the following equation: CT (μg/g) = [(A_450_ × V (mL) × 104)/A_450_% cm × P (g)]; A = absorbance, V = total extract volume, P = fresh sample weight, A% cm = 2592 (extinction coefficient). For AT, we ground ~500 mg of petals per sample in liquid nitrogen and extracted them in 5 mL of 1:99 (*v*/*v*) hydrochloric acid and methanol. We incubated the extract at 4 °C for 24 h and centrifuged it at 13,000 rpm for 20 min. We measured the absorbance (A) at A530 and calculated AT using the equation Q_AT_ = A_530_ × M^−1^; Q_AT_ = At amount, M = fresh weight (g). We performed each experiment in three to five replicates.

### 4.5. DNA Extraction and SSR Genotyping

Genomic DNA was extracted from young leaves using the CTAB method and quantified using a Nanodrop ND-1000 spectrophotometer (Thermo Scientific, Waltham, MA, USA). PCR was performed with the T100TM Thermal Cycler (BIO-RAD, Hercules, CA, USA) following the protocol of Zhou et al. [47]. Five polymorphic EST-SSR markers, selected from 48 markers previously identified as highly polymorphic1, were used for further analysis [21]. The amplicons were analyzed using 8% polyacrylamide gel electrophoresis (PAGE) at 300 V in a 1.0 × TBE buffer and visualized via silver staining. The sequence information of the five primer pairs is shown in Table S10.

### 4.6. Statistic Analysis

We calculated the maximum, minimum, mean, standard deviation, coefficient of variation, and mid-parent heterosis of PL, PD, FHH, FHD, RFL, DiD, and RFN. We applied hierarchical clustering analysis and principal component analysis to the phenotypic traits with the built-in functions of R 4.3.2 [48]. We performed Pearson correlation analysis and the “corrplot” package for plotting with R 4.3.2 [49]. We performed the Principal Coordinate Analysis (PCoA) with GenAlex 6.5 [50]. We estimated the Shannon–Wiener index of genetic diversity (*H*) for LAI, FT, DaD, OCL, and RFC traits and conducted the permutational multivariate analysis of variance (PERMANOVA, Mölndal, Sweden) with the “vegan” package in R 4.3.2 [51]. We conducted the Unweighted Pair Group Method with Arithmetic Mean (UPGMA) cluster analysis using Powermarker (V1.25) [52]. We performed the general linear model (GLM, K model) in TASSEL 4.3.65 for association analysis [53]. We considered marker-trait associations that were significant and extremely significant at *p* < 0.05. Population phenotypes for significant association loci were compared using Student’s *t*-test with the built-in functions of R 4.3.2 [48].

## 5. Conclusions

In this research, we examined the phenotypic and genotypic variation of 140 F_1_ hybrid progeny derived from the crossbreeding of two gerbera varieties with distinct flower types and colors. We assessed 12 crucial ornamental traits, including PL, PD, FHH, FHD, RFL, DiD, RFN, LAI, FT, DaD, OCL, and RFC. Additionally, we evaluated five parameters measured using a colorimeter and the CT and AT content of the ray floret. We employed cluster analysis, PCA, and correlation analysis to reveal the genetic patterns and diversity of the hybrid population. Furthermore, we utilized five EST-SSR markers to genotype the hybrid population, identifying 10 loci significantly associated with five traits, namely PL, RFL, LAI, OCL, and the b* of ray floret color. Our findings offer valuable insights and tools for the ornamental evaluation and genetic improvement of gerbera, laying the groundwork for future studies on the molecular mechanisms underlying the formation of significant floral traits.

## Data Availability

Data contained within the article.

## Supplementary Materials

The following supporting information can be downloaded at: https://www.mdpi.com/article/10.3390/ijms25010203/s1.

## References

[1] Carrodeguas-Gonzalez A., Zúñiga-Orozco A. (2021). Aplicación de herramientas moleculares para el mejoramiento genético de pasturas. Pastos Y Forrajes.

[2] Song X., Deng Z. (2013). Powdery Mildew Resistance in Gerbera: Mode of Inheritance, Quantitative Trait Locus Identification, and Resistance Responses. J. Am. Soc. Hortic. Sci..

[3] Teeri T.H., Elomaa P., Kotilainen M., Albert V.A. (2006). Mining plant diversity: Gerbera as a model system for plant developmental and biosynthetic research. Bioessays.

[4] Fu Y., Esselink G.D., Visser R.G.F., van Tuyl J.M., Arens P. (2016). Transcriptome Analysis of *Gerbera hybrida* Including in silico Confirmation of Defense Genes Found. Front. Plant Sci..

[5] Han M., Jin X., Yao W., Kong L., Huang G., Tao Y., Li L., Wang X., Wang Y. (2017). A Mini Zinc-Finger Protein (MIF) from *Gerbera hybrida* Activates the GASA Protein Family Gene, GEG, to Inhibit Ray Petal Elongation. Front. Plant Sci..

[6] Huang G., Han M., Jian L., Chen Y., Sun S., Wang X., Wang Y. (2020). An ETHYLENE INSENSITIVE3-LIKE1 Protein Directly Targets the GEG Promoter and Mediates Ethylene-Induced Ray Petal Elongation in *Gerbera hybrida*. Front. Plant Sci..

[7] Huang G., Han M., Yao W., Wang Y. (2017). Transcriptome analysis reveals the regulation of brassinosteroids on petal growth in *Gerbera hybrida*. PeerJ.

[8] Ren G., Li L., Huang Y., Wang Y., Zhang W., Zheng R., Zhong C., Wang X. (2018). GhWIP2, a WIP zinc finger protein, suppresses cell expansion in *Gerbera hybrida* by mediating crosstalk between gibberellin, abscisic acid, and auxin. New Phytol..

[9] Lin X., Huang S., Huang G., Chen Y., Wang X., Wang Y. (2021). 14-3-3 Proteins Are Involved in BR-Induced Ray Petal Elongation in *Gerbera hybrida*. Front. Plant Sci..

[10] Li L., Zhang W., Zhang L., Li N., Peng J., Wang Y., Zhong C., Yang Y., Sun S., Liang S. (2015). Transcriptomic insights into antagonistic effects of gibberellin and abscisic acid on petal growth in *Gerbera hybrida*. Front. Plant Sci..

[11] Kloos W.E., George C.G., Sorge L.K. (2004). Inheritance of the flower types of *Gerbera hybrida*. J. Am. Soc. Hortic. Sci..

[12] Kotilainen M., Helariutta Y., Mehto M., Pöllänen E., Albert V.A., Elomaa P., Teeri T.H. (1999). GEG Participates in the Regulation of Cell and Organ Shape during Corolla and Carpel Development in *Gerbera hybrida*. Plant Cell.

[13] Kumar R. (2013). Studies on Genetic Variability in Gerbera (*Gerbera jamesonii* Bolus Ex. Hooker F.). J. Hortic. Sci..

[14] Rymbai H., Verma V.K., Mawleiñ J., Hazarika S. (2023). Analysis of genetic divergence, principal component, correlation and path coefficient for quantitative traits of Gerbera (*Gerbera jamesonii*) in the north eastern region, India. Plant Genet. Resour. Charact. Util..

[15] Byrne D. (2006). Molecular Marker Use in Tree Fruit and Woody Ornamental Plant Breeding. Hortscience.

[16] Francia E., Tacconi G., Crosatti C., Barabaschi D., Bulgarelli D., Dall’Aglio E., Vale G. (2005). Marker assisted selection in crop plants. Plant Cell Tissue Organ Cult..

[17] Prajapati P., Singh A., Patel N.L., Singh D., Srivastav V. (2014). Evaluation of genetic diversity in different genotypes of Gerbera jamesonii Bolus using random amplified polymorphic DNA (RAPD) markers. Afr. J. Biotechnol..

[18] Saidi A., Hajkazemian M. (2023). Comparative assessment of ISSR, DAMD and RAPD markers for evaluation of genetic diversity of gerbera (*Gerbera jamesonii* Bolus ex Hooker f.) cultivars. Acta Agric. Slov..

[19] Gong L., Deng Z. (2010). EST-SSR markers for gerbera (*Gerbera hybrida*). Mol. Breed..

[20] Gong L., Deng Z. (2012). Selection and application of SSR markers for variety discrimination, genetic similarity and relation analysis in gerbera (*Gerbera hybrida*). Sci. Hortic..

[21] Zhou Y., Yin M., Abbas F., Sun Y., Gao T., Yan F., Li X., Yu Y., Yue Y., Yu R. (2022). Classification and Association Analysis of Gerbera (*Gerbera hybrida*) Flower Color Traits. Front. Plant Sci..

[22] Schlotterer C. (2004). The evolution of molecular markers—Just a matter of fashion?. Nat. Rev. Genet..

[23] Fu Y., van Silfhout A., Shahin A., Egberts R., Beers M., van der Velde A., van Houten A., van Tuyl J.M., Visser R.G.F., Arens P. (2017). Erratum to: Genetic mapping and QTL analysis of Botrytis resistance in *Gerbera hybrida*. Mol. Breed..

[24] Bhattarai K., Sharma S., Verma S., Peres N.A., Xiao S., Clark D.G., Deng Z. (2023). Construction of a genome-wide genetic linkage map and identification of quantitative trait loci for powdery mildew resistance in Gerbera daisy. Front. Plant Sci..

[25] Hârţa M., Clapa D., Cornea-Cipcigan M., Borsai O., Pop R., Cordea M.I. (2023). Multivariate Assessment of Genetic Relationships between Two Streptocarpus Cultivars and Their F1 Progenies Using Morphological Characteristics and SCoT Molecular Markers. Horticulturae.

[26] Rai R., Nguyen V.Y., Kim J.C. (2022). Variability analysis and evaluation for major cut flower traits of F_1_ hybrids in *Lilium brownii* var. colchesteri. J. Multidiscip. Sci..

[27] Yang Y., Wen C., Ma N., Zhao L. (2015). Heterosis and genetic analysis of branching in cut-flower chrysanthemums. Euphytica.

[28] Song X., Gao K., Fan G., Zhao X., Liu Z., Dai S. (2018). Quantitative Classification of the Morphological Traits of Ray Florets in Large-flowered Chrysanthemum. Hortscience.

[29] Song X., Xu Y., Gao K., Fan G., Zhang F., Deng C., Dai S., Huang H., Xin H., Li Y. (2020). High-density genetic map construction and identification of loci controlling flower-type traits in *Chrysanthemum* (*Chrysanthemum* × *morifolium* Ramat.). Hortic. Res..

[30] Wu X., Zhao X., Gao K., Tian Y., Zhang M., Anderson N.O., Dai S. (2023). Heterosis and Mixed Genetic Analysis of Flowering Traits in Cross Breeding of Day-Neutral Chrysanthemum (Asteraceae). Agronomy.

[31] Zhou Y., Abbas F., He J., Yan F., Wang Q., Yu Y., Yu R., Fan Y. (2022). Floral volatile chemical diversity in *Hedychium* F_1_ hybrid population. Ind. Crop. Prod..

[32] Wei X., Zhou Y., Abbas F., Yan F., Zou X., Yu Y., Gao T., He J., Wang Q., Yu R. (2023). Distant heteroploid hybridization improved *Hedychium* floral scent, floral color and morphologcal traits. Ind. Crop. Prod..

[33] Tanaka Y., Sasaki N., Ohmiya A. (2008). Biosynthesis of plant pigments: Anthocyanins, betalains and carotenoids: Harnessing plant biomass for biofuels and biomaterials. Plant J..

[34] Ng J., Freitas L.B., Smith S.D. (2018). Stepwise evolution of floral pigmentation predicted by biochemical pathway structure. Evolution.

[35] Zhao X., Zhang Y., Long T., Wang S., Yang J. (2022). Regulation Mechanism of Plant Pigments Biosynthesis: Anthocyanins, Carotenoids, and Betalains. Metabolites.

[36] Lu C., Li Y., Wang J., Qu J., Chen Y., Chen X., Huang H., Dai S. (2021). Flower color classification and correlation between color space values with pigments in potted multiflora chrysanthemum. Sci. Hortic..

[37] Yamamoto T. (2021). DNA Markers and Molecular Breeding in Pear and Other Rosaceae Fruit Trees. Hortic. J..

[38] Ibrahim A.K., Zhang L., Niyitanga S., Afzal M.Z., Xu Y., Zhang L., Zhang L., Qi J. (2020). Principles and approaches of association mapping in plant breeding. Trop. Plant Biol..

[39] Zhou Y., Ye Y., Zhu G., Xu Y., Tan J., Liu J. (2023). Diversity, classification, and EST-SSR-based association analysis of caladium ornamental traits. Physiol. Plant..

[40] Shi Z., Zhao W., Li Z., Kang D., Ai P., Ding H., Wang Z. (2022). Development and validation of SSR markers related to flower color based on full-length transcriptome sequencing in *Chrysanthemum*. Sci. Rep..

[41] Li S., Ji F., Hou F., Cui H., Shi Q., Xing G., Weng Y., Kang X. (2020). Characterization of Hemerocallis citrina Transcriptome and Development of EST-SSR Markers for Evaluation of Genetic Diversity and Population Structure of Hemerocallis Collection. Front. Plant Sci..

[42] Liu N., Cheng F. (2020). Association mapping for yield traits in Paeonia rockii based on SSR markers within transcription factors of comparative transcriptome. BMC Plant Biol..

[43] Yang Y., He R., Zheng J., Hu Z., Wu J., Leng P. (2020). Development of EST-SSR markers and association mapping with floral traits in *Syringa oblata*. BMC Plant Biol..

[44] Yan J., Warburton M., Crouch J. (2011). Association Mapping for Enhancing Maize (*Zea mays* L.) Genetic Improvement. Crop Sci..

[45] Sun X., Du Z., Ren J., Amombo E., Hu T., Fu J. (2015). Association of SSR markers with functional traits from heat stress in diverse tall fescue accessions. BMC Plant Biol..

[46] Gonnet J. (1998). Colour effects of co-pigmentation of anthocyanins revisited—1. A colorimetric definition using the CIELAB scale. Food Chem..

[47] Zhou Y., Wei X., Abbas F., Yu Y., Yu R., Fan Y. (2021). Genome-wide identification of simple sequence repeats and assessment of genetic diversity in *Hedychium*. J. Appl. Res. Med. Aromat. Plants.

[48] R Core Team (2023). R: A Language and Environment for Statistical Computing.

[49] Wei T., Simko V. (2021). R Package ‘Corrplot’: Visualization of a Correlation Matrix (Version 0.92). https://github.com/taiyun/corrplot.

[50] Peakall R., Smouse P.E. (2006). genalex 6: Genetic analysis in Excel. Population genetic software for teaching and research. Mol. Ecol. Notes.

[51] Oksanen J., Simpson G., Blanchet F., Kindt R., Legendre P., Minchin P., O’Hara R.B., Solymos P., Stevens M., Szoecs E. (2022). Vegan: Community Ecology Package. R Package Version 2.6-4. https://CRAN.R-project.org/package=vegan.

[52] Liu K., Muse S.V. (2005). PowerMarker: An integrated analysis environment for genetic marker analysis. Bioinformatics.

[53] Bradbury P.J., Zhang Z., Kroon D.E., Casstevens T.M., Ramdoss Y., Buckler E.S. (2007). TASSEL: Software for association mapping of complex traits in diverse samples. Bioinformatics.

