# Supplementary material for: Phenotypic Variation in Flower Color and Morphology in the Gerbera (Gerbera hybrida) F1 Hybrid Population and Their Association with EST-SSR Markers
Source: Int J Mol Sci. 2023 Dec 22;25(1):203. doi: 10.3390/ijms25010203 (PMC10779396; doi:10.3390/ijms25010203)
Supplement: Supplementary file 1 [file ijms-25-00203-s001.zip › ijms-2734450-supplementary.pdf]

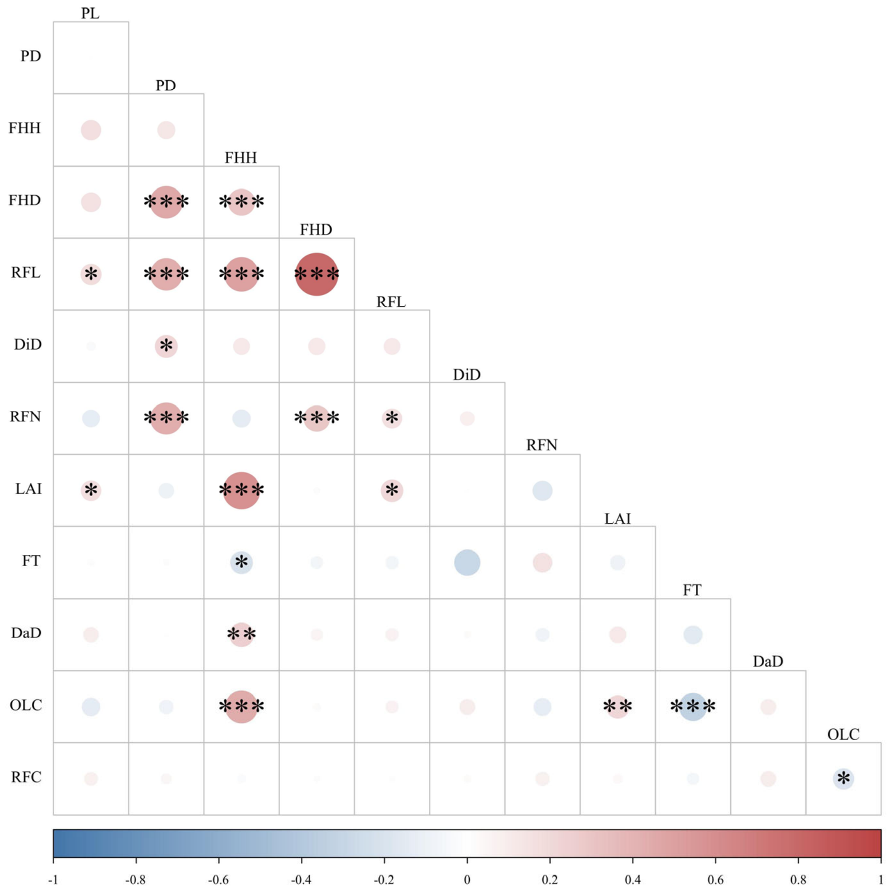

Figure S1. Pearson correlation analysis among 12 traits.

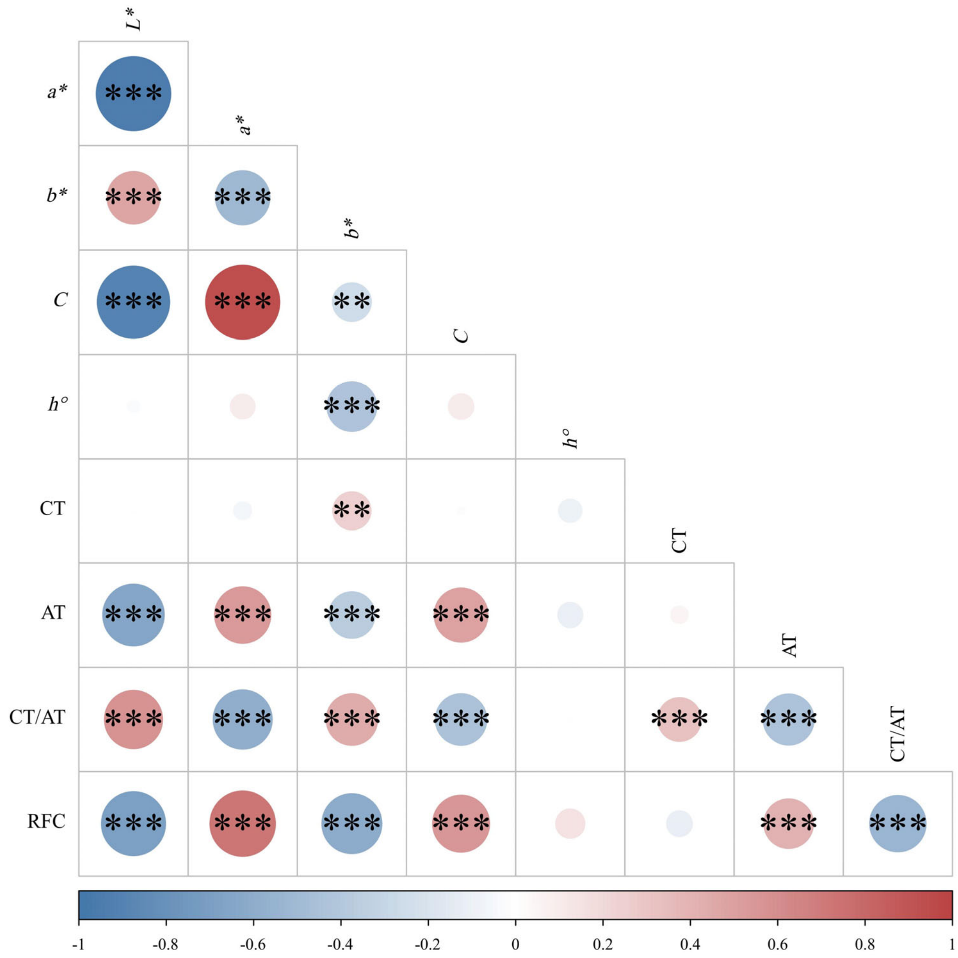

Figure S2. Pearson correlation analysis among *L**, *a**, *b**, *C*, *h*°, CT, AT, CT/AT and RFC.

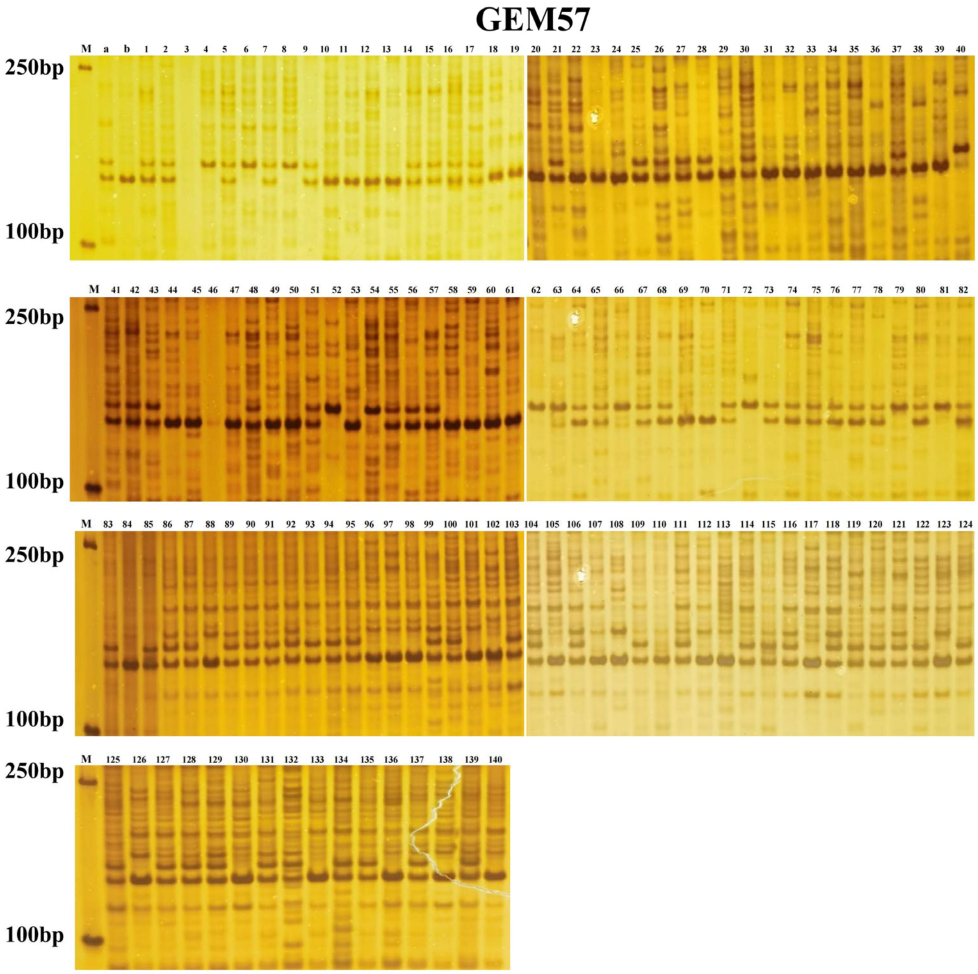

Figure S3. PAGE result of GEM57 primer.

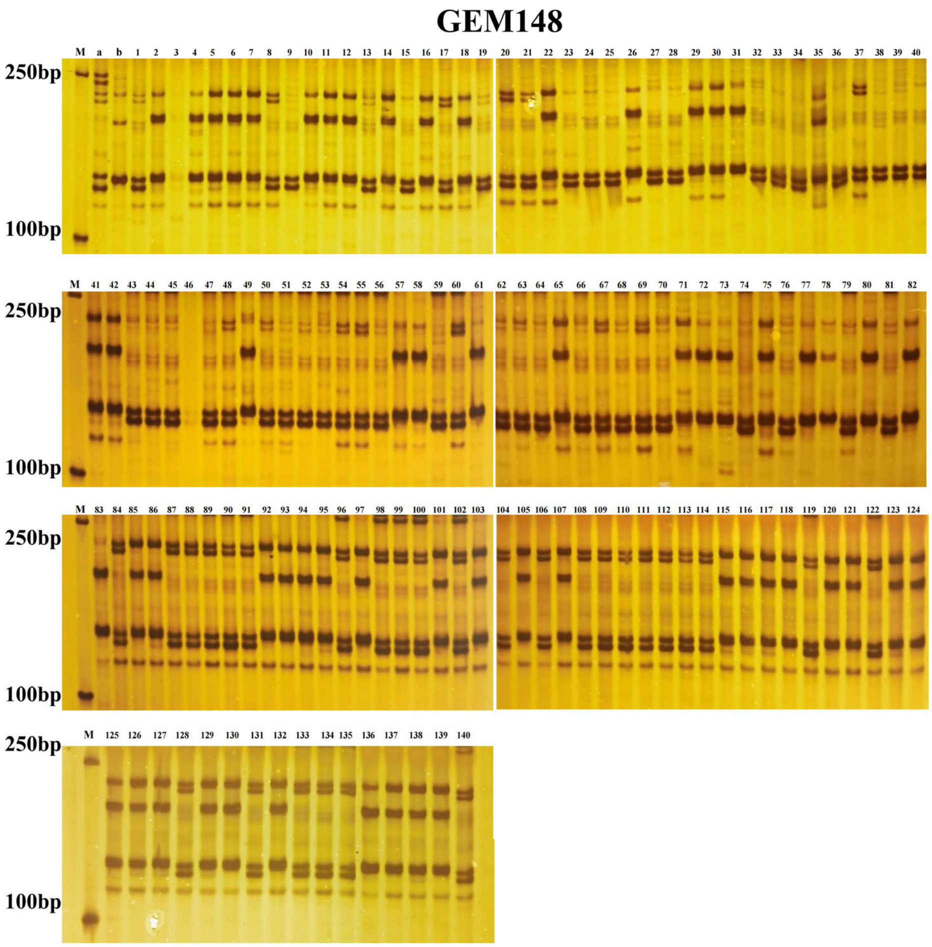

Figure S4. PAGE result of GEM148 primer.

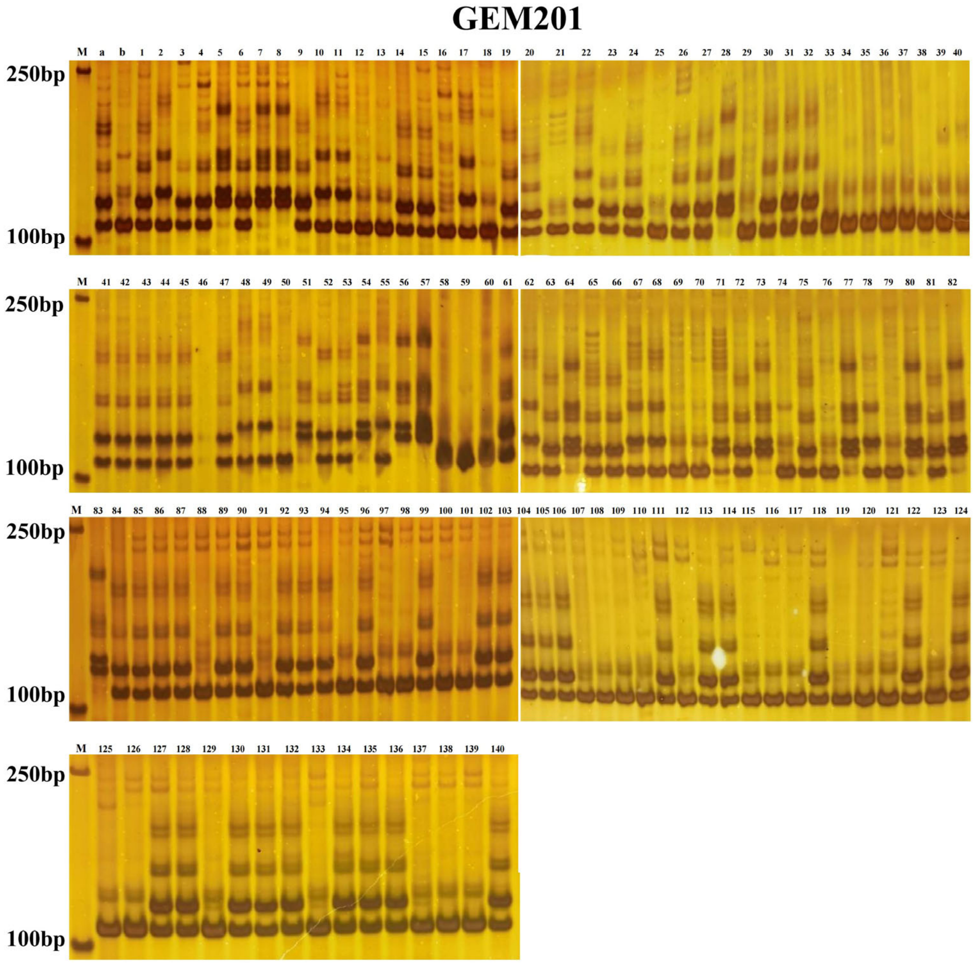

Figure S5. PAGE result of GEM201 primer.

GEM203

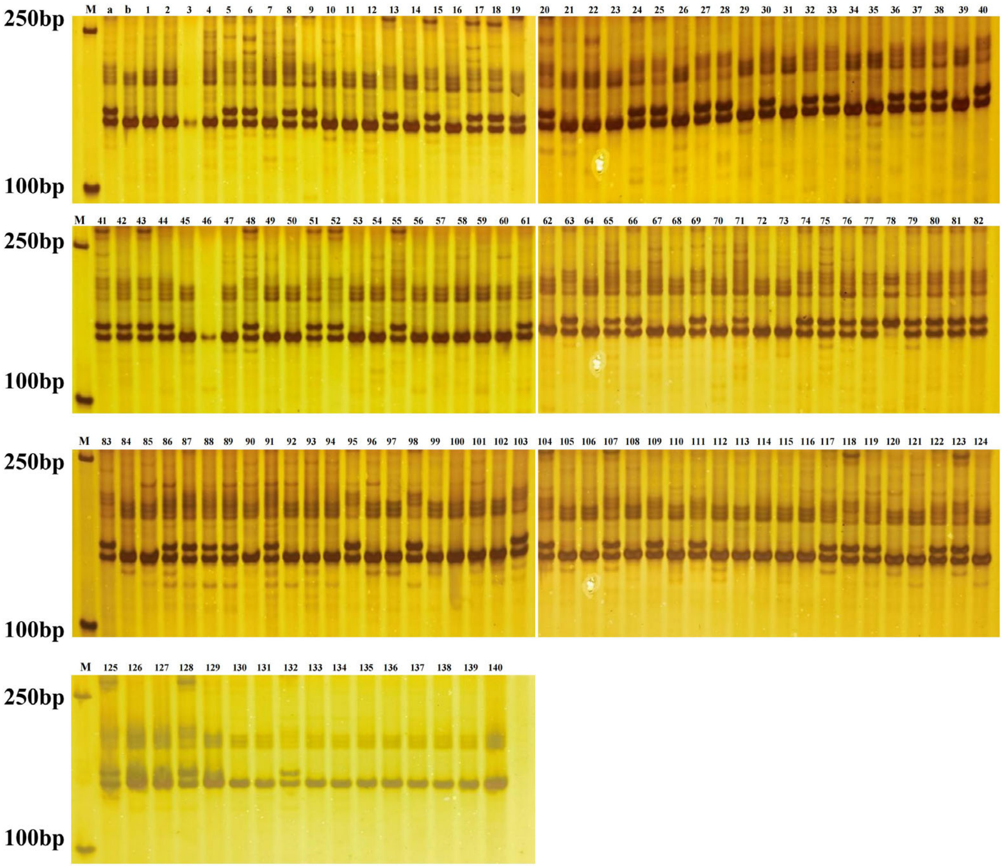

Figure S6. PAGE result of GEM203 primer.

P3-19

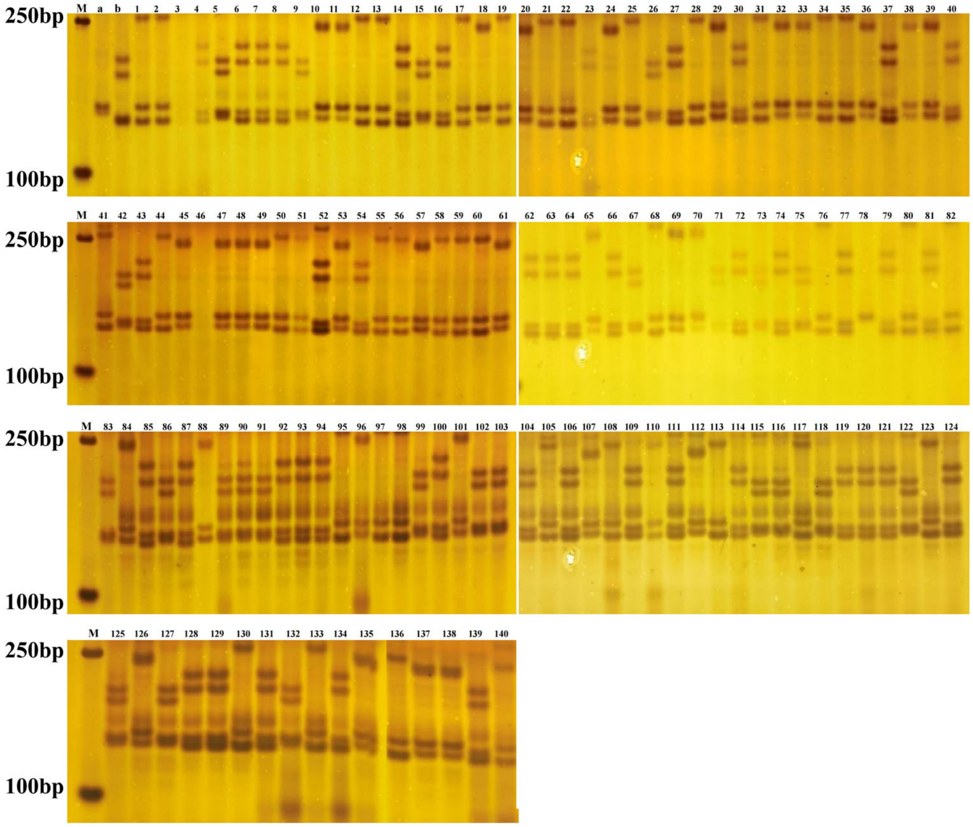

Figure S7. PAGE result of P3-19 primer.
